# Beta-Adrenergic Receptor 1 Selective Antagonism Inhibits Norepinephrine-Mediated TNF-Alpha Downregulation in Experimental Liver Cirrhosis

**DOI:** 10.1371/journal.pone.0043371

**Published:** 2012-08-20

**Authors:** Pedro Zapater, Isabel Gómez-Hurtado, Gloria Peiró, José Manuel González-Navajas, Irma García, Paula Giménez, Alba Moratalla, José Such, Rubén Francés

**Affiliations:** 1 The Biomedical Research Centre Network in the Area of Hepatic and Digestive Disorders (CIBERehd), Instituto de Salud Carlos III, Madrid, Spain; 2 Servicio de Farmacología Clínica, Hospital General Universitario, Alicante, Spain; 3 Unidad Hepática, Hospital General Universitario, Alicante, Spain; 4 Unidad de Investigación, Hospital General Universitario, Alicante, Spain; Hannover Medical University (MHH), Germany

## Abstract

**Background:**

Bacterial translocation is a frequent event in cirrhosis leading to an increased inflammatory response. Splanchnic adrenergic system hyperactivation has been related with increased bacterial translocation. We aim at evaluating the interacting mechanism between hepatic norepinephrine and inflammation during liver damage in the presence of bacterial-DNA.

**Animals and Methods:**

Forty-six mice were included in a 16-week protocol of CCl_4_-induced cirrhosis. Laparotomies were performed at weeks 6, 10, 13 and 16. A second set of forty mice injected with a single intraperitoneal dose of CCl_4_ was treated with saline, 6-hydroxidopamine, Nebivolol or Butoxamine. After 5 days, mice received *E. coli*-DNA intraperitoneally. Laparotomies were performed 24 hours later. Liver bacterial-DNA, norepinephrine, TNF-alpha, IL-6 and beta-adrenergic receptor levels were measured.

**Results:**

Bacterial-DNA translocation was more frequent in CCl_4_-treated animals compared with controls, and increased as fibrosis progressed. Liver norepinephrine and pro-inflammatory cytokines were significantly higher in mice with *vs* without bacterial-DNA (319.7±120.6 vs 120.7±68.6 pg/g for norepinephrine, 38.4±6.1 vs 29.7±4.2 pg/g for TNF-alpha, 41.8±7.4 vs 28.7±4.3 pg/g for IL-6). Only beta-adrenergic receptor-1 was significantly increased in treated *vs* control animals (34.6±7.3 *vs* 12.5±5.3, p = 0.01) and correlated with TNF-alpha, IL-6 and norepinephrine hepatic levels in animals with bacterial-DNA. In the second set of mice, cytokine levels were increased in 6-hydroxidopamine and Nebivolol (beta-adrenergic receptor-1 antagonist) treated mice compared with saline. Butoxamine (beta-adrenergic receptor-2 antagonist) didn’t inhibit liver norepinephrine modulation of pro-inflammatory cytokines.

**Conclusions:**

Beta-adrenergic receptor-1 mediates liver norepinephrine modulation of the pro-inflammatory response in CCl_4_-treated mice with bacterial-DNA.

## Introduction

Cirrhosis represents the end-stage of any chronic liver disease, characterized by the most advanced stage of fibrosis, distortion of the liver parenchyma associated with septae and nodule formation, altered blood flow and the potential development of liver failure at long term. Bacterial translocation (BT) is a common and recurrent event occurring in decompensated cirrhosis and constitutes the current pathogenic theory for the onset of bacterial infections in this setting [1]–[3]. Intestinal bacterial overgrowth, impairment in permeability of the intestinal mucosal barrier, and deficiencies in local host immune defences are the major mechanisms postulated to favour BT in cirrhosis [4], [5].

Experimental chronic liver damage is commonly associated with a several-fold increase of BT [6], [7]. Recently, our group showed that bacterial DNA (bactDNA) translocation incidence to mesenteric lymph nodes (MLNs) could be correlated with grade of fibrosis in mice treated with weight-controlled CCl_4_ increasing amounts during a 16-week study [8]. This was associated with changes in gut microbiota content favoring a pro-inflammatory TNF-α and IL-6 up-regulation. These cytokines had already been implicated in fibrosis progression in the past [9]–[11]. Promotion of hepatic fibrosis includes, though, many other players among which the sympathetic nervous system (SNS) is a prominent one [12]–[15].

Evidence of an enhanced SNS in patients with established decompensated cirrhosis has been reported in the past [16], [17]. SNS plays an immunosuppressive role, mainly through norepinephrine (NE), in chemotaxis and phagocytosis [18]–[20], which are relevant activities in host response against Gram-negative microorganims. In fact, it has recently been shown in rats with CCl_4_-induced cirrhosis that hyperactivity of the splanchnic SNS contributes to the translocation of *E coli* but not *S aureus* to MLNs and extraintestinal sites. Also, splanchnic sympathectomy reduced bacterial translocation to MLN in ascitic cirrhotic rats from 45% to 17% [21]. Related with all this, propanolol decreases the rates of bacterial overgrowth and translocation in cirrhotic rats with ascites [22], and the use of beta-blockers has been reported to prevent SBP in patients with cirrhosis and ascites, independent of haemodynamic response [23].

Since we have demonstrated the increasing incidence of BT, mainly by *E. coli*, during the induction of liver cirrhosis in the mouse experimental model of chemically-induced liver damage, we hypothesize that SNS must be hyperactivated, not only favouring fibrosis but also a progressively augmented BT rate in these animals. This study will provide insight on interactions between bactDNA translocation, NE and inflammation activity at different stages in the liver, the main organ of inflammation in cirrhosis, as progressive damage is induced by CCl_4_ administration in mice and may offer rationale for new therapeutic strategies aim at preventing BT in cirrhosis.

## Methods

### Ethics Statement

Animals received care according to the criteria outlined in the Guide for the Care and Use of Laboratory Animals. The study was approved by the Animal Research Committee of Universidad Miguel Hernandez (Alicante, Spain) with approval number HA-RFG-002-09.

### Animals and Study Design

Female Balb/c 8-week old mice (Harlan, Barcelona, Spain) were included in a 16-week study protocol for induction of chronic liver damage (Protocol I) or in a 2-week protocol for chemical sympathectomy (Protocol II). Mice were caged at a constant room temperature of 21°C and exposed to a 12∶12 light/dark cycle.

### Protocol I

#### Induction of liver damage and laparotomies

**Figure 1:**
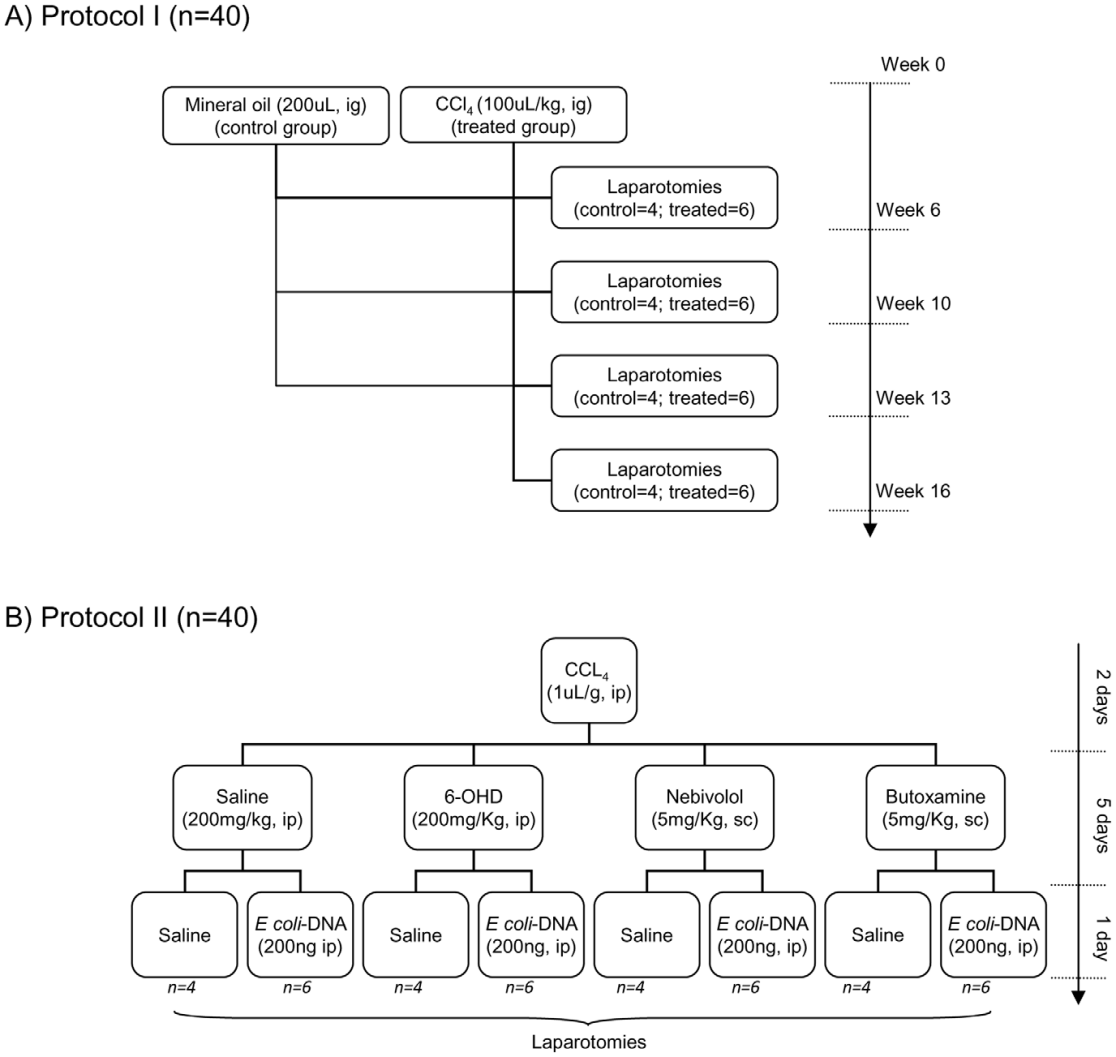
Design of study protocols. (A) Study Protocol I. Balb/c mice were included in a 16-week protocol for induction of liver cirrhosis by oral administration of CCl_4_. A subgroup of animals (six CCl_4_-treated and four control mice) was subjected to laparotomy at different study weeks to follow progression of fibrosis, inflammation, bactDNA translocation and NE hepatic levels. CCl_4_: carbon tetrachloride; ig.: intragastrically. (B) Study Protocol II. CCl_4_-treated Balb/c mice were distributed to receive saline, 6-OHD for sympathetic denervation, Nebivolol for beta-adrenergic receptor (ADRB)-1 blockade, or Butoxamine for ADRB-2 blockade. Within each group, animals were further distributed to receive saline or *E. coli*-DNA. Laparotomies were performed in all subgroups. CCl_4_: carbon tetrachloride; ip: intraperitoneally; 6-OHD: 6-hydroxydopamine; sc: subcutaneously;

Mice weighting 18–20 g were fed standard rodent chow and were treated with 0.25 mmol/L phenobarbital in tap water along the study protocol. After a 4-week housing in those conditions, liver damage was induced by intragastrical CCl_4_ administration and laparotomies were performed at 6, 10, 13 and 16 weeks in a subgroup of treated mice (n = 6/week) and at 0, 6, 10, 13 and 16 weeks in a subgroup of control animals (n = 4/week) (Figure 1A), as previously described [8].

#### Sample collection

The liver was perfused *in situ* with 6 ml of Hanks balanced salt solution (HBSS) without Ca^2+^ and Mg^2+^ at 37°C at a rate of 1.5 ml/min. This was followed by perfusion with 12 ml of HBSS containing 0.02% collagenase and 100 mM CaCl_2_ solution at the same perfusion rate. The liver was then removed and rinsed with 3 mL of HBSS. A liver fraction was immediately preserved with EDTA 1 mM and sodium metabisulfite 4 mM and frozen to avoid catecholamine degradation. Histopathological, microbiological and molecular studies were performed in all collected liver samples. Spleen was also collected.

#### Histological analysis

Liver biopsy specimens between 10 to 15 mm in size were fixed in buffered formalin and embedded in paraffin. Histologic changes were first evaluated by routine hematoxilin and eosin (H&E) in four-micrometer thick sections. We estimated the severity of hepatic fibrosis and architectural distortion with the connective tissue stain Masson trichrome. The amount of fibrosis was blindly assessed semiquantitatively based on the Ishak score [24] by a single senior pathologist (GP), with a conventional light microscope (Olympus BX50, Barcelona, Spain).

#### Gene expression analysis of pro-fibrogenic markers

Total cellular RNA was isolated from 20–30 mg of frozen liver preserved in RNAlater RNA stabilization reagent (Qiagen, Hilden, Germany) disrupted by sonication (Hielscher UP100H Ultrasonic Processor, Teltow, Germany) and handled according to QuantiTect SYBRGreen RT-PCR kit (Qiagen) manufacturer’s instructions. Quantitative PCR was used to determine expression of profibrogenic Procollagen α-1(1) (ProCol-1), tumour growth factor beta (TGF-β)-1, tissue inhibitor of metalloproteinase (TIMP)-1 matrix metalloproteinase (MMP)-2 and adrenergic receptor beta (ADRB)-1, -2 and -3 genes as previously described [8]. Gene expression levels were normalized to β2-microglobulin. Primer-pair sequences used in the study can be followed in Table S1.

#### Identification of bacterial DNA

MLNs and livers were disrupted by sonication in a Tissue Lyser II (Qiagen). Total DNA was isolated by using the QIAamp DNA Tissue kit (Qiagen) according to the manufacturer’s instructions. Bacterial DNA presence was identified by polymerase chain reaction (PCR) followed by partial nucleotide sequencing of 16SrRNA gene according to the methodology described elsewhere [25].

#### Measurement of catecholamine and cytokine levels in control and treated mice livers

The 3-CAT Research ELISA kit (LDN, Nordhorn, Germany) and the Mouse Quantikine ELISA kits (R&D Systems, Minneapolis, MN) were used to determine norepinephrine (NE), and TNF-alpha and IL-6 levels respectively, in disrupted livers from control and treated mice, according to manufacturer’s instructions. Protein hepatic levels of ADRB-1, ADRB2 and ADRB3 were also evaluated by ELISA kits (USCNK, Wuhan, PR China) according to manufacturer’s instructions. Measurements were read by triplicate in a Tecan Sunrise automated microplate reader (Männedorf, Switzerlad).

### Protocol II

A second set of female Balb/c 8-week old mice (n = 40) was included for additional experiments on NE-mediated effects over the inflammatory response. To resemble the chemically-induced liver damage of the first protocol, mice received an intraperitoneal single dose of CCl_4_ (1uL/g body weight) [26]. Forty-eight hours later, a blood sample was obtained by venopunction of the tail vein to confirm liver damage through alanine aminotransferase (ALT) and total billirrubin (TBIL) levels using Mammalian Liver Profile for VetScan VS2 (Abaxis, Union City, CA). Mice were then injected either saline, 6-hydroxidopamine (6-OHD) (Sigma, Spain) (200 mg/Kg) [27] intraperitoneally for chemical sympathectomy, Nebivolol or Butoxamine (Sigma) (5 mg/Kg every 12 h, each) subcutaneously for the selective blockade of ADRB1 and ADRB2, respectively. After 5 days, animals from all groups received *E. coli* bactDNA (200 ng) intraperitoneally. A subgroup of animals remained without *E. coli* injection as controls. Laparotomies were performed at 24 hours after *E. coli*-DNA injection (Figure 1B). Liver and MLNs were collected as described above.

BactDNA was evaluated in all specimens, and measurement of catecholamines and cytokines in the liver was performed as described above.

### Statistical Analysis

Continuous variables are reported as mean±standard deviation and categorical variables as frequency or percentages. Statistical differences between groups were analyzed using the chi-square test for categorical data and the Mann-Whitney U test for quantitative data. Statistical differences between 3 or more groups were analyzed using the Kruskal–Wallis test. Bivariate correlations between continuous variables were calculated using the Spearman test. Multiple comparisons were analyzed using pairwise comparisons using Mann-Whitney U test and Bonferroni correction to determine if the post-hoc tests were significant. All reported *P* values are 2-sided, and *P* values lower than.05 are considered to indicate significance. All calculations were performed using the SPSS 19.0 software (SPSS, Inc, Chicago, IL).

## Results

### Characteristics of Animals and Progressive Liver Injury

A total of 62 animals were included in the protocol for induction of cirrhosis (Protocol I). Forty of them were weekly treated with CCl_4_ and 22 mice constituted the control group. Sixteen animals died during liver damage induction (treated group). Cause of death was CCl_4_ toxicity and liver insufficiency. None of the control animals died during the study protocol. According to previous results [8], animals were grouped in basal and two protocol stages based on fibrosis degree: one from the beginning of liver bactDNA evaluation (week 6) to week 10, defined by mild fibrosis (Stage-1), and a second one from week 13 to week 16 characterized by severe fibrosis (Stage-2). Characteristics of control and treated mice at all these stages can be followed in Table 1.

**Table 1:** Characteristics of CCl_4_-treated mice included in the study at different Stages.

	Basal (week 0)	Stage 1 (weeks 6–10)	Stage 2 (weeks 13–16)
	*control (n = 6)*	*control (n = 8)*	*control (n = 8)*
Body weight (gr)	19,80±0,30	22,17±0,74	23,28±0,55
Liver weight (gr)	0,92±0,12	0,89±0,06	1,04±0,05
Spleen weight (gr)	0,08±0,03	0,10±0,02	0,11±0,02
TGFB-1 mRNA Rel Exp	1,20±0,14	1,21±0,18	1,28±0,15
mmp2 mRNA Rel Exp	1,14±0,12	1,36±0,14	1,29±0,12
Proc-1 mRNA Rel Exp	1,12±0,14	1,15±0,16	1,28±0,19
TIMP-1 mRNA Rel Exp	1,11±0,13	1,24±0,25	1,27±0,23
		*CCl4 (n = 12)*	*CCl4 (n = 12)*
Body weight (gr)		21,48±1,23	21,48±1,44
Liver weight (gr)		1,28±0,15*	1,35±0,15* $
Spleen weight (gr)		0,13±0,03*	0,15±0,01* $
Accum CCl_4_ dosage (uL)		631,50±60,65	1348,26±103,50
TGFB-1 mRNA Rel Exp		4,84±2,52*	29,81±4,31* $
mmp2 mRNA Rel Exp		6,85±3,51*	41,50±4,24* $
Proc-1 mRNA Rel Exp		10,20±5,20*	42,40±6,15* $
TIMP-1 mRNA Rel Exp		14,16±8,56*	45,90±7,82* $
Fibrosis Grade (Ishak)	0±0	2,50±0.92 (2–3)	5,12±0,54 (4–5)

* *p<0,05 compared with Basal and Stage control group*

$ *p<0,05 compared with Stage 1*

Gene markers of fibrosis were significantly increased from Stage-1 when compared with levels in non-treated mice. Their highest relative expression and the most severe liver fibrosis, as evaluated by the Ishak Scale, corresponded with Stage-2. Accumulated dose of CCl_4_ correlated with relative expression of all markers of fibrosis (*vs* TGF-B, r = 0.815; *vs* MMP-2, r = 0.794; *vs* ProCol-1, r = 0.817; *vs* TIMP-1, r = 0.893; *p*<0.001 in all cases).

### Evidence of bactDNA Translocation in Liver of Treated Animals

MLN bactDNA translocation was more frequently observed in treated animals compared with control mice (14/24, 58.3% *vs* 3/22, 13.6%, *p* = 0.01). MLN bactDNA sequencing analysis identified *Escherichia coli* in 11 samples (9 treated, 2 control), *Streptococcus pneumoniae* in 3 samples (2 treated, 1 control), and *Staphylococcus aureus*, *Shigella flexneri* and *Campylobacter jejuni* in 1 sample of treated mice. Interestingly, bactDNA was also found in disrupted liver samples of treated animals (10/24, 41.6%) and *Escherichia coli* (8 samples), *Streptococcus pneumoniae* (1 sample) and *Shigella flexneri* (1 sample) were identified from treated mice. *Staphylococcus aureus* was identified in the single bactDNA-positive liver from control mice. All bacteria identifications in liver samples corresponded with those in MLNs, either from control or treated animals.

BactDNA translocation rate in control mice was not increased along the study weeks. Among treated animals, percentage of bactDNA translocation in the liver was significantly incremented at Stage-2 (66% liver bactDNA) compared with Stage-1 (25% liver bactDNA, p = 0.04), as can be followed in Table 2.

**Table 2:** Presence of bactDNA in MLNs and livers of control and CCl_4_-treated mice at different Stages.

	Control mice (n = 22)		Treated mice (n = 24)		
	MLNs	Liver	MLNs	Liver	
Basal (week 0)	0/6 (0%)	0/6 (0%)	–	–	
Stage 1 (weeks 6–10)	1/8 (12,5%)	1/8 (12,5%)	4/12 (33%)*	3/12 (25%)*	
Stage 2 (weeks 13–16)	1/8 (12,5%)	0/8 (0%)	10/12 (83%)*	8/12 (66%)*	

* *p<0,05 compared with the stage control group; MLNs: mesenteric lymph nodes*

### Hepatic NE and Inflammatory Cytokine Levels during Induction of Cirrhosis

In Stage-1, liver NE, TNF-α and IL-6 levels were significantly increased in treated animals compared with the basal and the stage control mice (Table 3). Regarding Stage-2, a further statistically significant increment in hepatic NE levels was observed compared with treated animals in Stage-1, whereas TNF-α and IL-6 showed no further differences. A positive correlation between hepatic NE levels and TGF-beta gene expression levels was present in CCl4-treated animals (r = 0.772, p = 0.01). No correlations between fibrosis progression, as determined either by profibrogenic gene expression levels or Ishak scores, and proinflammatory cytokines were observed.

**Table 3:** Liver NE, cytokine and ADRB levels in control and CCl4-treated mice in basal and protocol Stages.

		Basal (week 0)	Stage 1 (weeks 6–10)	Stage 2 (weeks 13–16)
*Control mice*		*n = 6*	*n = 8*	*n = 8*
	Norepinephrine (pg/g)	33,86±19,70	30,12±22,40	38,72±12,47
	TNF-alpha (pg/g)	21,04±5,36	19,61±5,28	20,35±4,87
	IL-6 (pg/g)	17,69±3,56	16,43±3,38	16,92±3,89
	ADRB1 (ng/g)	13,93±5,69	14,79±4,97	12,53±5,27
	ADRB2 (ng/g)	21,30±7,60	22,50±10,46	19,63±4,69
	ADRB3 (ng/g)	18,15±5,90	20,06±10,11	19,12±8,42
*CCL_4_ mice*			*n = 12*	*n = 12*
	Norepinephrine (pg/g)		105,75±66.73*	334,74±134,6* $
	TNF-alpha (pg/g)		34,08±10.62*	28,67±4.93*
	IL-6 (pg/g)		38,99±15.2*	32,88±8.17*
	ADRB1 (ng/g)		13,84±7,48	34,64±7,31* $
	ADRB2 (ng/g)		18,07±5,87	17,73±4,51
	ADRB3 (ng/g)		19,11±4,60	18,04±4,95

* *p<0,05 compared with basal and the stage control group;*

$ *p<0,05 compared with CCl4-mice in Stage 1*

*Stage 1: liver bactDNA≤25%; Stage 2: liver bactDNA>65%; ADRB: beta-adrenergic receptor*

**Figure 2:**
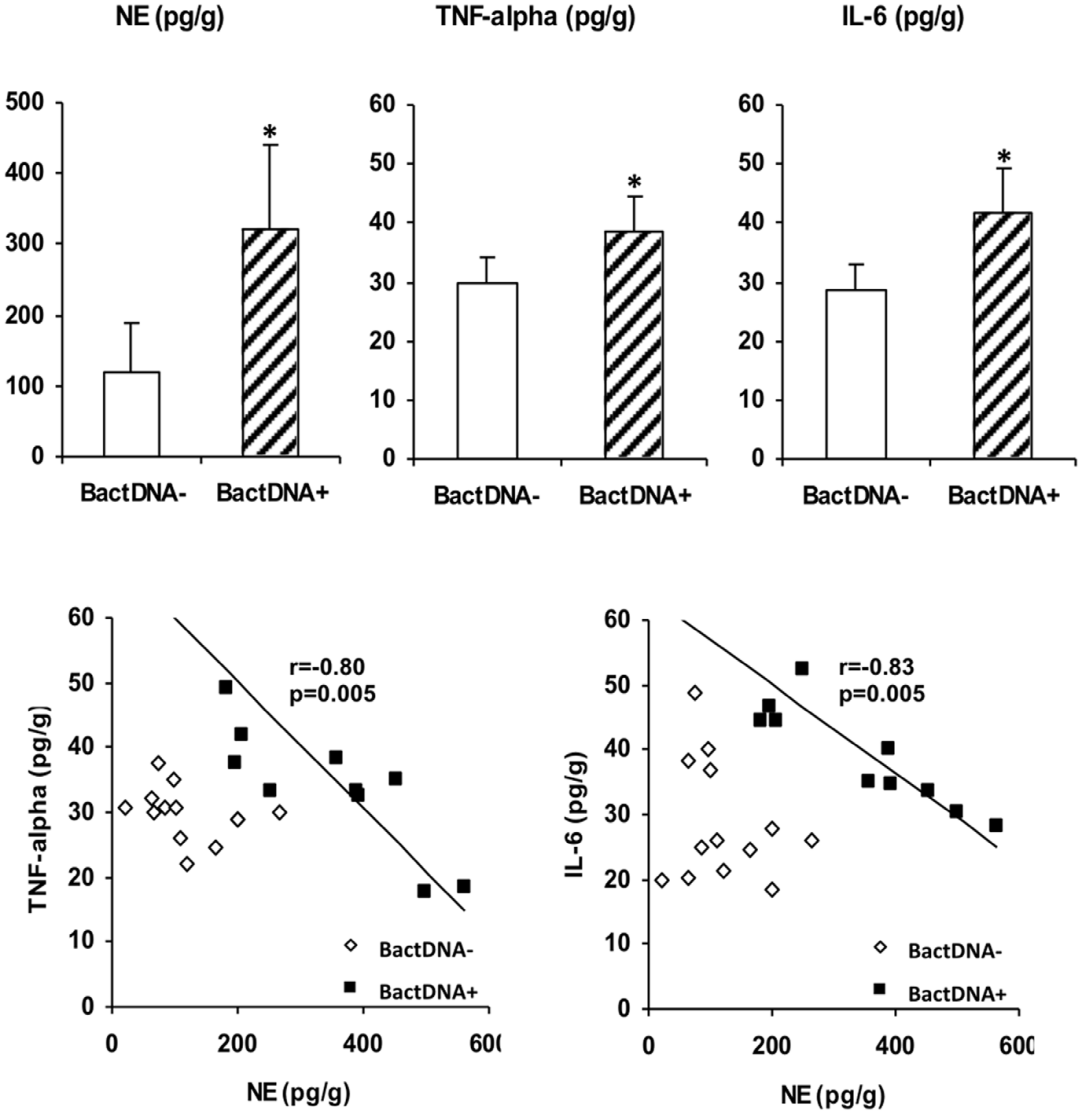
Inflammatory and sympathetic activities in the liver. (A) Liver levels of NE, TNF-alpha and IL-6 in CCl_4_-treated mice according to bactDNA translocation into liver. *p<0.05 compared with bactDNA-negative CCl_4_-treated mice. (B) Correlations between liver NE, TNF-alpha and IL-6 in CCl_4_-treated mice according to bactDNA translocation into liver. r: Spearman’s rank correlation coefficient in bactDNA+ mice; NE: norepinephrine; bactDNA: bacterial DNA.

NE and inflammatory cytokine levels were significantly higher in the liver of treated mice with bactDNA translocation compared with treated animals without bactDNA (Figure 1). An inverse correlation was found between NE and all cytokines studied in the liver of treated animals with bactDNA, whereas no correlation was observed between the hepatic levels of NE and inflammatory cytokines in animals without bactDNA translocation into liver (Figure 2).

**Figure 3:**
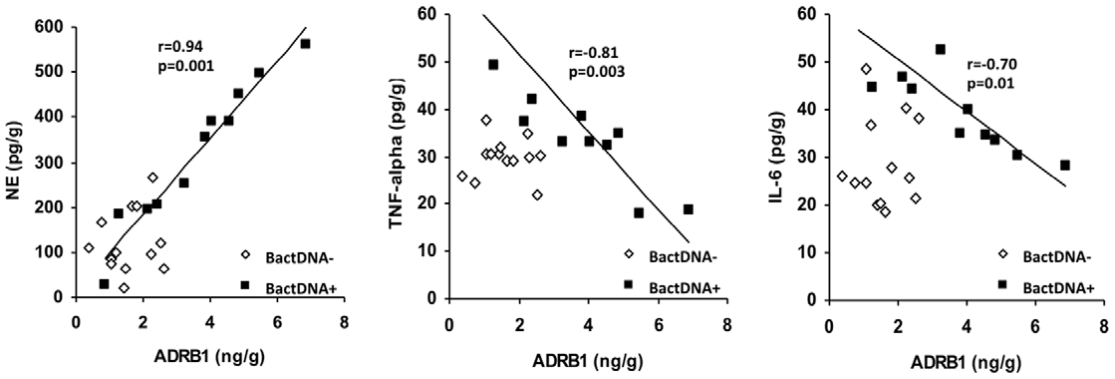
Interaction between inflammatory and sympathetic activities in the liver. Correlations between ADRB1 levels and NE, TNF-alpha and IL-6 levels in the liver of CCl_4_-treated mice according to bactDNA translocation. r: Spearman’s rank correlation coefficient in bactDNA+ mice; NE: norepinephrine; ADRB1: beta-1 adrenergic receptor; bactDNA: bacterial DNA.

We then evaluated beta-adrenergic receptor levels in the liver of animals at different stages of cirrhosis induction (Table 3). ADRB1 was the only receptor showing a statistically significant increment between treated and control animals at Stage 2. Besides, ADRB1 protein levels correlated with the two cytokines studied and with NE hepatic levels in the subgroup of animals with liver bactDNA translocation, whereas this correlation was not found in animals without bactDNA (Figure 3). ADRB1 increment was also present at mRNA level, showing an inverse correlation with TNF-alpha (r = −0.75, p = 0.01), IL-6 (r = −0.72, p = 0.01) and NE (r = 0.88, p = 0.001) in animals with bactDNA. No correlations were observed for ADRB2 or ADRB3 in any case (Table S2).

### NE Modulates the Liver Pro-inflammatory Response to bactDNA through ADRB1

To confirm the interaction between NE and the inflammatory response in the presence of bactDNA in the liver, a second protocol involving chemical sympathectomy and the use of selective ADRB1 (Nebivolol) and ADRB2 (Butoxamine) antagonists was run in mice treated with CCl_4_ (Figure 1B). After 6-OHD-induced denervation, saline, Nebivolol or Butoxamine treatment in a CCl_4_ environment, animals received *E. coli*-DNA to evaluate the inflammatory response to this bacterial antigen. Forty mice were included in this protocol. Hepatic injure was confirmed in all cases (ALT>2000 U/L; TBIL>0.5 mg/dL). mRNA expression of profibrogenic genes, as markers of liver damage, didn’t show statistically significant differences between treatments with saline, 6-OHD and ADRB1 or ADRB2 antagonists (Table S3). NE levels in the liver were undetectable in all mice treated with 6-OHD. PCR and sequencing analysis confirmed bactDNA presence from *E. coli* in all mice.

**Figure 4:**
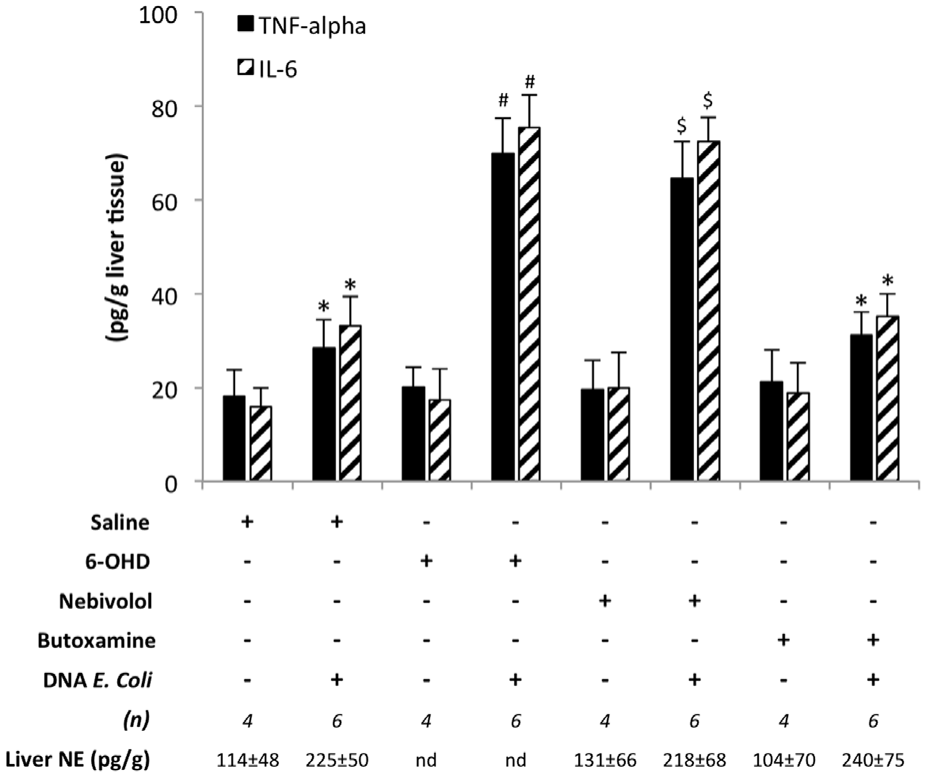
Liver inflammatory levels after different treatments. Levels of TNF-alpha and IL-6 in the liver of mice undergoing different experimental conditions. All mice were pretreated with an intraperitoneal single dose of CCl_4_ (1uL/g body weight) to generate liver damage. After 2 days, animals were grouped for a 5-day treatment with saline, 6-OHD (200 mg/Kg, intraperitoneal), Nebivolol (5 mg/Kg every 12 h, subcutaneous) or Butoxamine (5 mg/Kg every 12 h, subcutaneous). Then, animals received *E. coli* DNA (200ng, intraperitoneal). A subgroup of animals remained without *E. coli* DNA injection as controls. *p<0,05 compared with all *E. coli* DNA-negative conditions; ^#^p<0,05 compared with the rest of conditions, except Nevibolol+/*E. coli* DNA+; ^$^p<0,05 compared with the rest of conditions, except 6-OHD+/*E. coli* DNA+; 6-OHD: 6-hydroxidopamine; NE: norepinephrine.

Administration of *E. coli*-DNA induced a significant upregulation of TNF-α and IL-6 in all studied conditions. However, both the ablation of SNS and the selective blockade of ADRB1 with Nebivolol prior to administration of *E. coli* bactDNA induced further significantly higher levels of TNF-α and IL-6 in the liver than mice treated with saline or with butoxamine prior to administration of *E. coli*-DNA (Figure 4). On the other hand, no differences were observed between chemically sympathectomized animals and those receiving the selective ADRB1 antagonist. However, statistically significant differences in the hepatic levels of TNF-α and IL-6 were present between 6-OHD and butoxamine treated mice. These data suggest that NE is implicated in tempering down the pro-inflammatory response to bactDNA and that ADRB1 is selectively implicated in mediating this process.

## Discussion

The present study demonstrates that hepatic NE exerts an immunomodulatory effect over the pro-inflammatory activity observed in the presence of liver bactDNA translocation and that this effect requires ADRB1 in CCl_4_-induced liver damage.

Experimental BT has been correlated in the last years both with an increased inflammatory response [7] and with an SNS hyperactivation [21] in advanced decompensated cirrhosis with ascites. BactDNA translocation into MLN can be found in preascitic cirrhotic rats associated with an enhanced cytokine profile at a systemic level through the activation of immune cells at the hepatic draining lymph nodes [28]. Also, our group showed the increasing incidence of bactDNA translocation in mice during progression of CCl_4_-induced liver damage and its association with the decrease of anti-inflammatory gut microbiota content [8].

**Figure 5:**
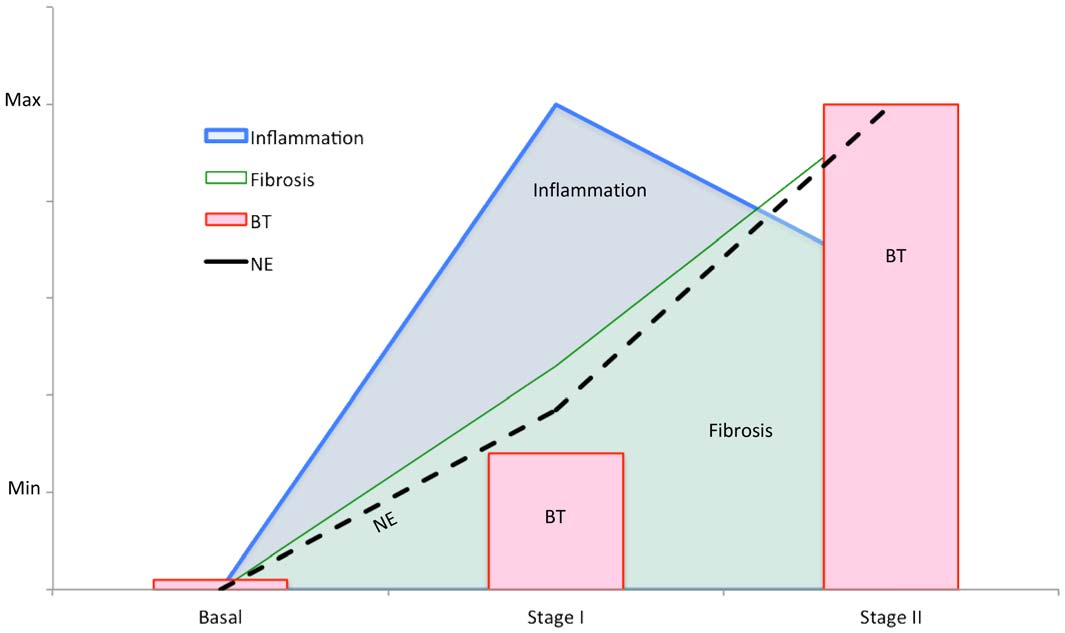
Integration of different processes along study weeks. Illustration of the temporal evolution on the interaction between fibrosis, bacterial translocation and pro-inflammatory and sympathetic activities in the experimental model of CCl_4_-induced liver damage. BT: bacterial translocation; NE: norepinephrine.

The staged analysis of liver damage induction has allowed us to evaluate the temporal evolution of inflammatory and sympathetic activities, fibrosis progression and bactDNA translocation. TNF-α and IL-6 had already been implicated in fibrosis progression in the past. In fact, if we consider only the beginning (basal) and the end (Stage 2, severe fibrosis) of the induction protocol, a correlation can be found between fibrosis and proinflammatory cytokines. However, intermediate stage results reveal an earlier activation of the inflammatory machinery that may be setting the favorable conditions for fibrosis progression. NE release would then be triggered to temper down the initial pro-inflammatory response and, consequently, BT would be facilitated (Figure 5).

In the mice model of CCl_4_-induced liver damage, hepatic NE, TNF-α and IL-6 levels were significantly higher in bactDNA+ *vs* bactDNA– animals. Interestingly, hepatic levels of NE correlated directly with TGF-β gene expression and inversely with TNF-alpha and IL-6 concentrations in animals with bactDNA (Figure 1). Considering these results, NE would not only act as a profibrogenic agent, as described [12], [15] but also as a modulator of excessive pro-inflammatory response in the liver. This regulation of hepatic pro-inflammatory response might be facilitating bactDNA translocation into liver. In fact, NE has been reported to inhibit LPS-derived TNF-α and IL-6 levels in human whole blood [29].

ADRB are widely expressed on immune cells and play a role in modulating macrophagic function. In fact, ADRB1/ADRB2−/− mice exhibit increased TNF-alpha and decreased IL-10 serum levels in response to LPS challenge relative to wild type animals [30]. In our study, ADRB1 levels in the liver of CCl_4_-treated mice were significantly increased, they correlated directly with hepatic NE levels and inversely with TNF-α and IL-6 in animals with liver bactDNA translocation (Figure 2). These data suggest the involvement of ADRB1in NE modulation of the inflammatory response to bactDNA translocation. In fact, levels of TNF-α and IL-6 were significantly increased only after a selective blockade of ADRB1 with Nebivolol (Figure 3). These data confirm first, an NE-inflammation crosstalk in the presence of bactDNA, and second, the prominent role of ADRB1 as receptor in the NE modulation of hepatic TNF-α and IL-6 levels. NE signalling through ADRB1 is a well-known mechanism in which adenylate cyclase is activated resulting in a cyclic adenosine monophosphate (cAMP) intracellular increase. cAMP binds protein kinase A (PKA) and their interaction downregulates nuclear factor kappa B (NF-kB) signalling, therefore decreasing pro-inflammatory cytokine secretion [31].

From a clinical standpoint, it has been reported the beneficial effects of non-selective beta-blockers in patients with cirrhosis that have recently been associated with a reduction in community-acquired SBP, independently of the hemodynamic response [23], [32]. However, the use of beta blockers has been associated with poor survival in patients with cirrhosis and refractory ascites [33]. NE is a vasoconstrictor agent that can be used as an alternative to terlipressin in the treatment of hepatorenal syndrome [34]. The possible effect of NE or terlipressin administration on an increased BT rate and subsequent hemodynamic changes is yet to be elucidated in this setting. Although our data suggest that NE administration and the subsequent ADRB1 activation would facilitate increased rates of BT, specific studies would be necessary to ascertain whether administration of terlipressin could have the same effect, as terlipressin acts through different receptor mechanisms. Considering all this data, it would be interesting that new studies using beta-blockers or NE evaluate bactDNA translocation events in decompensated cirrhosis. Data would also support the startup of studies on novel therapeutic strategies based on ADRB antagonists aimed at preventing BT in this setting. In fact, a recent study has demonstrated a beneficial effect of a beta-1 blocker on survival over septic rats through preservation of gut barrier function [35].

In conclusion, the present investigation demonstrates a specific interaction between hepatic NE and the pro-inflammatory response in mice with CCl_4_-induced liver damage and bactDNA presence that is mediated through ADRB1.

## Supporting Information

Table S1 **Primer-pair sequences used in the study.**
(DOC)

Table S2 **Correlation scores between ADRB2 and ADRB3 with TNF-alpha, IL-6 and NE.**
(DOC)

Table S3 **mRNA expression of profibrogenic genes in animals treated with saline, 6-OHD, ADRB1 and ADRB2 antagonists.**
(DOC)
