# Supplementary material for: Beta-Adrenergic Receptor 1 Selective Antagonism Inhibits Norepinephrine-Mediated TNF-Alpha Downregulation in Experimental Liver Cirrhosis
Source: PLoS One. 2012 Aug 20;7(8):e43371. doi: 10.1371/journal.pone.0043371 (PMC3423372; doi:10.1371/journal.pone.0043371)
Supplement: Table S1 — Primer-pair sequences used in the study. (DOC) [file pone.0043371.s001.doc]

| **Supplementary Table 1.** |  | |  |
| --- | --- | --- | --- |
|  |  | |  |
| **Gene** | | **Sequence** | |
| β-2 microglobulina | | GTGACCCTGGTCTTTCTGGT | |
|  |  | ATCCCAGTAGACGGTCTTGG | |
|  | |  | |
| Procollagen *α*1 (I) | | TCCGGCTCCTGCTCCTCTTA | |
|  |  | GTATGCAGCTGACTTCAGGGATGT | |
|  | |  | |
| Matrix Metalloproteinase 2 (MMP-2) | | CCGAGGACTATGACCGGGATAA | |
|  |  | CTTGTTGCCCAGGAAAGTGAAG | |
|  | |  | |
| Tumour Growth Factor beta (TGF-*β*) | | CCTGAGTGGCTGTCTTTTGA | |
|  |  | CAACCCAGGTCCTTCCTAAA | |
|  | |  | |
| Tissue Inhibitor of Metalloproteinase 1 (TIMP-1) | | TCCTCTTGTTGCTATCACTGATAGCTT | |
|  |  | CGCTGGTATAAGGTGGTCTCGTT | |
|  | |  | |
| 16S rRNA | | AGAGTTTGATCATGGCTCAG | |
|  |  | ACCGCGACTGCTGCTGGCAC | |
|  | |  | |
| Beta-adrenergic receptor (ADRB)1 | | CCGAACCCTGCAACCTGTCGT | |
|  |  | GCGCGCGTCAGCAAACTCTG | |
|  | |  | |
| ADRB2 | | GAGCGCCAGACCACGACGTC | |
|  |  | TGTGACTGGCCCCAAACGGC | |
|  | |  | |
| ADRB3 | | GAAAGGTGTGTGGACTGGGCAGT | |
|  |  | GGGCGTCCGACCACAAAGCC | |
|  | | | |
