# Supplementary material for: Beta-Adrenergic Receptor 1 Selective Antagonism Inhibits Norepinephrine-Mediated TNF-Alpha Downregulation in Experimental Liver Cirrhosis
Source: PLoS One. 2012 Aug 20;7(8):e43371. doi: 10.1371/journal.pone.0043371 (PMC3423372; doi:10.1371/journal.pone.0043371)
Supplement: Table S2 — Correlation scores between ADRB2 and ADRB3 with TNF-alpha, IL-6 and NE. (DOC) [file pone.0043371.s002.doc]

| **Supplementary Table 2.** | | | |  | | | |  |
| --- | --- | --- | --- | --- | --- | --- | --- | --- |
|  | | | |  | | | |  |
|  | ADRB1 | | ADRB2 | | | ADRB3 | | |
|  | bactDNA - | bactDNA + | bactDNA - | | bactDNA + | bactDNA - | bactDNA + | |
| NE | r=0,38; p=0,1 | r=0,94; p=0,001 | r=0,34; p=0,2 | | r=0,31; p=0,5 | r=0,15; p=0,8 | r=0,25; p=0,6 | |
| TNF-alpha | r=-0,16; p=0,7 | r=-0,81; p=0,003 | r=-0,26; p=0,6 | | r=-0,24; p=0,6 | r=-0,18; p=0,8 | r=-0,17; p=0,8 | |
| IL-6 | r=-0,30; p=0,5 | r=-0,70; p=0,01 | r=-0,32; p=0,5 | | r=-0,34; p=0,3 | r=-0,15; p=0,8 | r=-0,21; p=0,7 | |
| *ADRB: beta-adrenergic receptor; bactDNA: bacterial DNA; NE: norepinephrine* | | | | | |  |  | |
