# Supplementary material for: Beta-Adrenergic Receptor 1 Selective Antagonism Inhibits Norepinephrine-Mediated TNF-Alpha Downregulation in Experimental Liver Cirrhosis
Source: PLoS One. 2012 Aug 20;7(8):e43371. doi: 10.1371/journal.pone.0043371 (PMC3423372; doi:10.1371/journal.pone.0043371)
Supplement: Table S3 — mRNA expression of profibrogenic genes in animals treated with saline, 6-OHD, ADRB1 and ADRB2 antagonists. (DOC) [file pone.0043371.s003.doc]

| **Supplementary Table 3.** | | | | | | |  | | | | | | | |
| --- | --- | --- | --- | --- | --- | --- | --- | --- | --- | --- | --- | --- | --- | --- |
|  | | | | | | |  | | | | | | | |
| **Protocol II** | Saline | | | 6-OHD | | | | Nebivolol | | | Butoxamine | | |  |
| TGFB-1 mRNA Rel Exp | 19,22 | ± | 6,15 | 23,49 | ± | 11,42 | | 25,02 | ± | 10,60 | 20,48 | ± | 9,12 |  |
| mmp2 mRNA Rel Exp | 41,90 | ± | 8,22 | 45,05 | ± | 12.46 | | 46,16 | ± | 7,25 | 42,80 | ± | 7,25 |  |
| Proc-1 mRNA Rel Exp | 45,48 | ± | 10,10 | 48,19 | ± | 9.84 | | 50,04 | ± | 8,65 | 46,30 | ± | 8,42 |  |
| TIMP-1 mRNA Rel Exp | 50,11 | ± | 7,65 | 52,49 | ± | 10.16 | | 54,80 | ± | 12,10 | 51,52 | ± | 8,50 |  |
